# Navigating Novel Uncertainties of COVID-19: The Rise of the JN.1 Variant

**DOI:** 10.7759/cureus.51497

**Published:** 2024-01-02

**Authors:** Ibraheem Altamimi, Ibrahim M Alabdulkarim, Abdullah S Alhumimidi, Mohammed A Albabtain, Mohamad-Hani Temsah

**Affiliations:** 1 College of Medicine, King Saud University, Riyadh, SAU; 2 Pediatric Intensive Care Unit, College of Medicine, King Saud University, Riyadh, SAU; 3 Evidence-Based Healthcare and Knowledge Translation Research, King Saud University, Riyadh, SAU

**Keywords:** covid-19 vaccine, covid-19 pandemic, epidemiology and public health, jn.1, omicron, covid-19

## Abstract

In the shadow of the coronavirus disease 2019 (COVID-19) pandemic, the emergence of the JN.1 variant highlights the need for continued vigilance. This Editorial examines the characteristics of JN.1, derived from BA.2.86, and how it affects global public health. Despite its mutation on the spike protein and rapid spread, there has been no increase in disease severity, particularly in terms of ICU admissions, as evidenced by data from the World Health Organization (WHO) and the Centers for Disease Control and Prevention (CDC). We emphasize the importance of continued surveillance, vaccine adaptation, and public health preparedness while advocating for a balanced response to effectively manage the post-pandemic era. It reflects on the resilience built through vaccination efforts and the need for international cooperation to navigate the way forward in the face of additional severe acute respiratory syndrome coronavirus 2 (SARS-CoV-2) variants.

## Editorial

In the still-evolving landscape of the coronavirus disease 2019 (COVID-19) pandemic, the emergence of the JN.1 severe acute respiratory syndrome coronavirus 2 (SARS-CoV-2) variant marks another chapter of much-needed vigilance and adaptation. Descended from BA.2.86, JN.1 is an Omicron variant that has captured the attention of the global health authorities. What sets JN.1 apart is a critical mutation on its spike protein, a key factor in the virus's ability to infect human cells, featuring a critical mutation, S:L455S, in its spike protein. This mutation marks a significant divergence from the HK.3 and other "FLip" variants, which have the S:L455F mutation. The study highlights that S:L455S contributes to JN.1's increased immune evasion capabilities, potentially explaining its higher effective reproductive number compared to other variants. Moreover, JN.1 demonstrates robust resistance to monovalent XBB.1.5 vaccine sera, underscoring its potential as a highly immune-evading variant. This mutation potentially signifies changes in the virus's behavior, including its transmissibility, and the effectiveness of current vaccines against it [1].

As we navigate these uncertain healthcare challenges, it is necessary to balance caution with perspective. While JN.1's rapid emergence and genetic distinctiveness warrant close monitoring, there is currently no evidence suggesting a drastic increase in severity as compared to previous variants [1-3]. Nevertheless, this does not undermine the necessity for vigilance and readiness. The potential consequences of the mutation in the spike protein extend to the validity of vaccines and the virus's capacity to evade immune detection. This necessitates ongoing investigation and adjustment of our immunization approaches to guarantee their efficacy against emerging variants [1]. The narrative surrounding JN.1 encompasses not only a novel variant but also our continuous struggle against COVID-19 and other mutations in respiratory viruses that have the potential to induce extensive infections, whether at the regional or international level, is evident [2,3].

This emphasizes the critical need for resilient worldwide surveillance networks, expeditious exchange of genomic information, and an ongoing dedication to public health protocols. In addition, the emergence of JN.1 highlights the significance of preventative measures, including hygiene practices and non-pharmacological interventions, such as face masks, as well as vaccinations, which are dynamic processes that may require periodic updates to keep up with the evolution of the virus [2,3]. Above all, JN.1 reminds us of the unpredictability of the pandemic. It teaches us the value of resilience and adaptability in the face of new challenges. As we continue to learn more about JN.1, let us stay informed, vigilant, and proactive, reinforcing the lessons we have learned throughout this pandemic crisis, to safeguard our communities against emerging threats. This editorial aims to explore the JN.1 variant's rapid rise and its implications while emphasizing the importance of continuous vigilance and adaptation in our response to COVID-19.

**Figure 1:**
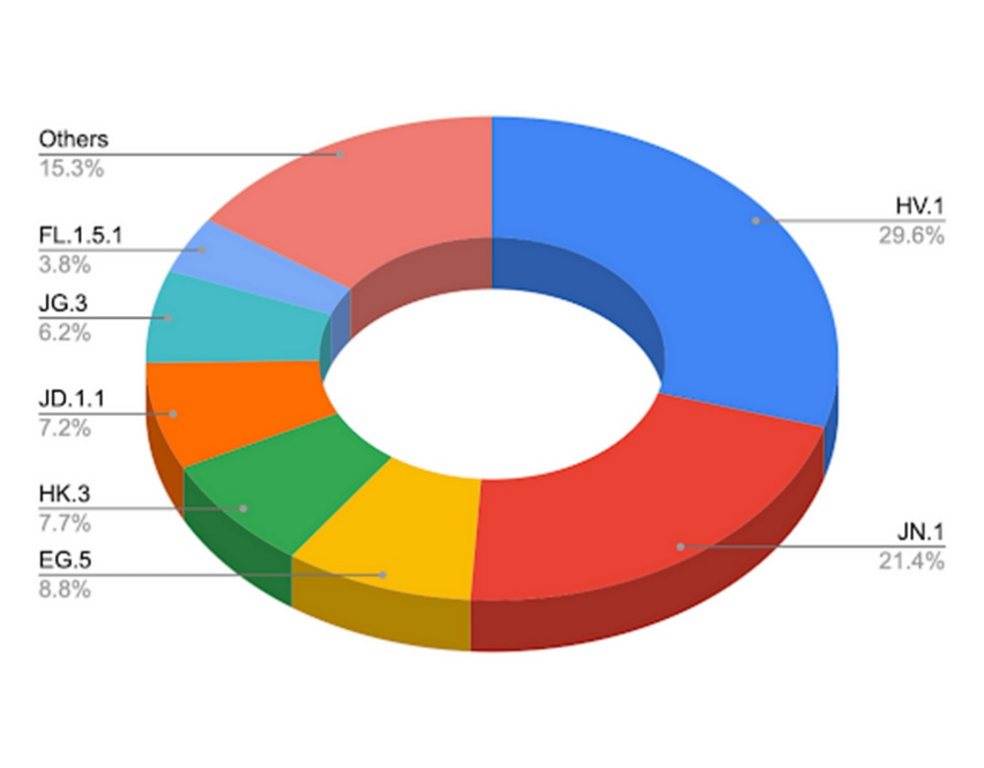
Prevalence of SARS-CoV-2 variants in the United States

The distribution of SARS-CoV-2 variants in the United States in December 2023 is captured in Figure 1, which emphasizes the significant prevalence of the JN.1 variant [2]. As illustrated, JN.1 now accounts for over one-fifth of all COVID-19 cases, signaling a significant shift in the viral landscape. These data not only underscore the variant's rapid rise but also the critical need for ongoing genomic surveillance to track such changes in real time. As the pandemic continues to unfold, these figures serve as a stark reminder of the virus's capacity for mutation and the importance of maintaining robust public health strategies to mitigate its impact. In a recent development, the Saudi Public Health Authority reported a significant local spread of the JN.1 variant, accounting for 36% of cases [4]. Notably, this rise has not led to an increase in critical care admissions, suggesting that while the variant may be more transmissible, it does not necessarily correlate with more severe illness. This aligns with the global understanding that variants, while concerning, do not always result in heightened severity of disease.

The Saudi authority's observations lend weight to the argument that while vigilance remains paramount, panic is unwarranted. It also highlights the importance of considering regional data when shaping the global response to new variants. The steady ICU admissions during rising variant cases suggest growing viral resilience, aided by widespread immunity from vaccinations and past infections. Moreover, effective vaccination strategies and strong healthcare systems are key to this stability, underscoring a holistic pandemic management approach [4]. As the situation unfolds, the insights from Saudi Arabia's experience with JN.1 offer a silver lining: the pandemic, while persistent, is becoming an entity that we are learning to live with and manage, without resorting to the emergency measures that marked its onset. It underscores the need for a measured approach that balances precaution with practicality, ensuring that public health strategies are adaptable and proportionate to the risk posed [5-7].

The JN.1 variant's emergence underlines COVID-19's evolving threat, with a disconnect between case numbers and severe outcomes due to widespread immunity. To effectively navigate this challenge, it's crucial to maintain vigilance and enhance global collaboration. Practical measures like sharing real-time genomic surveillance data, coordinating vaccine distribution, and jointly developing treatment protocols exemplify the necessary collaborative efforts for a proactive global response to ongoing and future health crises.
